# Research Progress of Long Non-Coding RNA GAS5 in Malignant Tumors

**DOI:** 10.3389/fonc.2022.846497

**Published:** 2022-06-28

**Authors:** Guohong Lin, Tianzhun Wu, Xing Gao, Ziqin He, Wenwei Nong

**Affiliations:** ^1^ Department of General Surgery, Affiliated Minzu Hospital of Guangxi Medical University, Nanning, China; ^2^ Oncology Medical College, Guangxi Medical University, Nanning, China

**Keywords:** growth arrest specific transcript 5, long non-coding RNA, neoplasms, neoplasm invasiveness, prognosis

## Abstract

With completing the whole genome sequencing project, awareness of lncRNA further deepened. The growth arrest-specific transcript 5 (GAS5) was initially identified in growth-inhibiting cells. GAS5 is a lncRNA (long non-coding RNA), and it plays a crucial role in various human cancers. There are small ORFs (open reading frames) in the exons of the GAS5 gene sequence, but they do not encode functional proteins. In addition, GAS5 is also the host gene of several small nucleolar RNAs (snoRNA). These snoRNAs are believed to play a suppressive role during tumor progression by methylating ribosomal RNA (rRNA). As a result, GAS5 expression levels in tumor tissues are significantly reduced, leading to increased malignancy, poor prognosis, and drug resistance. Recent studies have demonstrated that GAS5 can interact with miRNAs by base-pairing and other functional proteins to inhibit their biological functions, impacting signaling pathways and changing the level of intracellular autophagy, oxidative stress, and immune cell function *in vivo*. In addition, GAS5 participates in regulating proliferation, invasion, and apoptosis through the above molecular mechanisms. This article reviews the recent discoveries on GAS5, including its expression levels in different tumors, its biological behavior, and its molecular regulation mechanism in human cancers. The value of GAS5 as a molecular marker in the prevention and treatment of cancers is also discussed.

## Introduction

The human genome is composed of coding genes and non-coding genes. There are about 20,000 protein-coding genes, but these genes only represent less than 2% of the human genome. Non-coding RNAs (ncRNAs) account for the other 98% and have been called “dark matter” by researchers, contrary to the dogma, they cannot translate proteins (1). However, ncRNAs are not a “noise” of genetic transcription; on the contrary, increasing studies have shown that ncRNAs are widely involved in the process of cell physiology, for example, in regulating gene expression, maintaining genome stability, and contributing to the formation of the complexity of the organism (2).

Based on their length, RNAs can be classified into short RNAs (sRNAs, < 200 nt) or long ncRNAs (lncRNAs, > 200 nt). Short RNAs include micro RNAs (miRNAs, 22 nt), small interfering RNAs (siRNAs, 22 nt), small nuclear RNAs (snRNAs, 100–300 nt), PIWI-associated RNAs (piRNAs, 27 nt), and small nucleolar RNAs (snoRNAs, 70 nt) and other types of sRNAs (2, 3). Brannan et al. (4) isolated and sequenced the first lncRNA, H19, from the human genome in 1990. One of the first observations was that H19 does not encode a protein, unlike traditional mRNA. Numerous lncRNAs are transcribed by RNA polymerase II (RNA Pol II) and can be classified as exonic, intronic, overlapping, or intergenic lncRNAs according to the location of the gene (5). Depending on the function of the lncRNA, they can be further classified as cis-acting lncRNAs that affect local gene expression or trans-acting lncRNAs that operate far away from the transcription site (6). Furthermore, lncRNA can accelerate targeted protein binding to specific genomic loci, influence the bioactivity of protein-binding partners, and regulate gene expression as a small nuclear RNA precursor (7).

## GAS5

**Figure 1:**
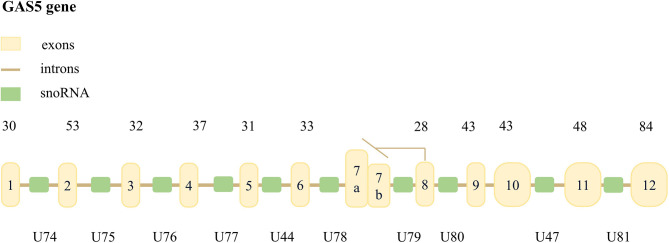
The structure of GAS5 in the human genome. The yellow boxes represent the 12 exons of human GAS5, the brown line represents 11 introns, and the green boxes represent 10 snoRNAs. The number above the gene indicates the length of the exon, and the bottom of the gene is the symbol of snoRNA. It is pointed out that alternative splicing of exon 7 may transcribe two mature lncRNAs, named GAS5a and GAS5b, respectively. The former is 39 bases longer than the latter.

Schneider et al. (8) initially found GAS5 in a mouse cell model of negative growth regulation. GAS5 is a lncRNA with 651 nucleotides encoded by a sequence located at 1q25.1. The GAS5 sequence includes 12 exons, 11 introns, and 10 identical small ORFs on the corresponding introns snoRNA, but this sequence does not encode a protein (9, 10). GAS5 can be transcribed into two mature RNAs, GAS5a and GAS5b, using the selective 5’-splice donor site on the 7th exon (**Figure 1**) (11). GAS5 transcripts contain a 7-nucleotide 5’oligopyrimidine tract, a translation initiation point, and a short 5 ‘UTR. Hence, it is considered a member of the 5’TOP gene family (12).

More than 20 splice variants of GAS5 have been detected, with two variants, “Full-Length” (FL) and “Clone 2” (C2), having been shown to have different effects on the cell proliferation phenotype of neuroblastoma. Research on molecule mechanism shows that silencing GAS5 increased the number of p53 phosphorylation. At the same time, another candidate gene, BRCA1(Breast Cancer 1), was identified as co-activating with p53, and their downstream co-acting target GADD45A (Growth Arrest and DNA Damage Inducible Alpha) was further detected. GADD45A was shown to induce cell cycle arrest and promote apoptosis. Although the supplementation of the FL splice variant of GAS5 reduces p53 levels, the supplementation of the C2 variant does no effect on p53 and drives apoptosis. Therefore, the above studies suggest that GAS5 splicing variants may cause tumor suppressor or tumor-promoting effect of GAS5 in different cancers (13).

Recent studies supported that GAS5 regulates cell cycle progression and exerts biological functions. Active cyclin dependent kinases 4/6 (CDK4/6) release part of the restriction of E2F1 by phosphorylation of the retinoblastoma protein (Rb), which inhibits cell proliferation as a tumor suppressor (14). c-MYC is considered to be a proto-oncogene. After translation, c-MYC is suppressed by GAS5, which can stop the G1-S transition by upregulating the expression levels of CDK inhibitors and p21 (15). GAS5 has a hormone response element mimic (HREM) sequence, which can bind to nuclear steroid receptors to accelerate cell apoptosis, improve the effect of chemotherapy drugs and DNA damage from radiation, and other *in vitro* apoptosis-stimulating factors (16).

## The Tumor Suppressor Mechanism of GAS5 in Various Types of Cancer

**Figure 2:**
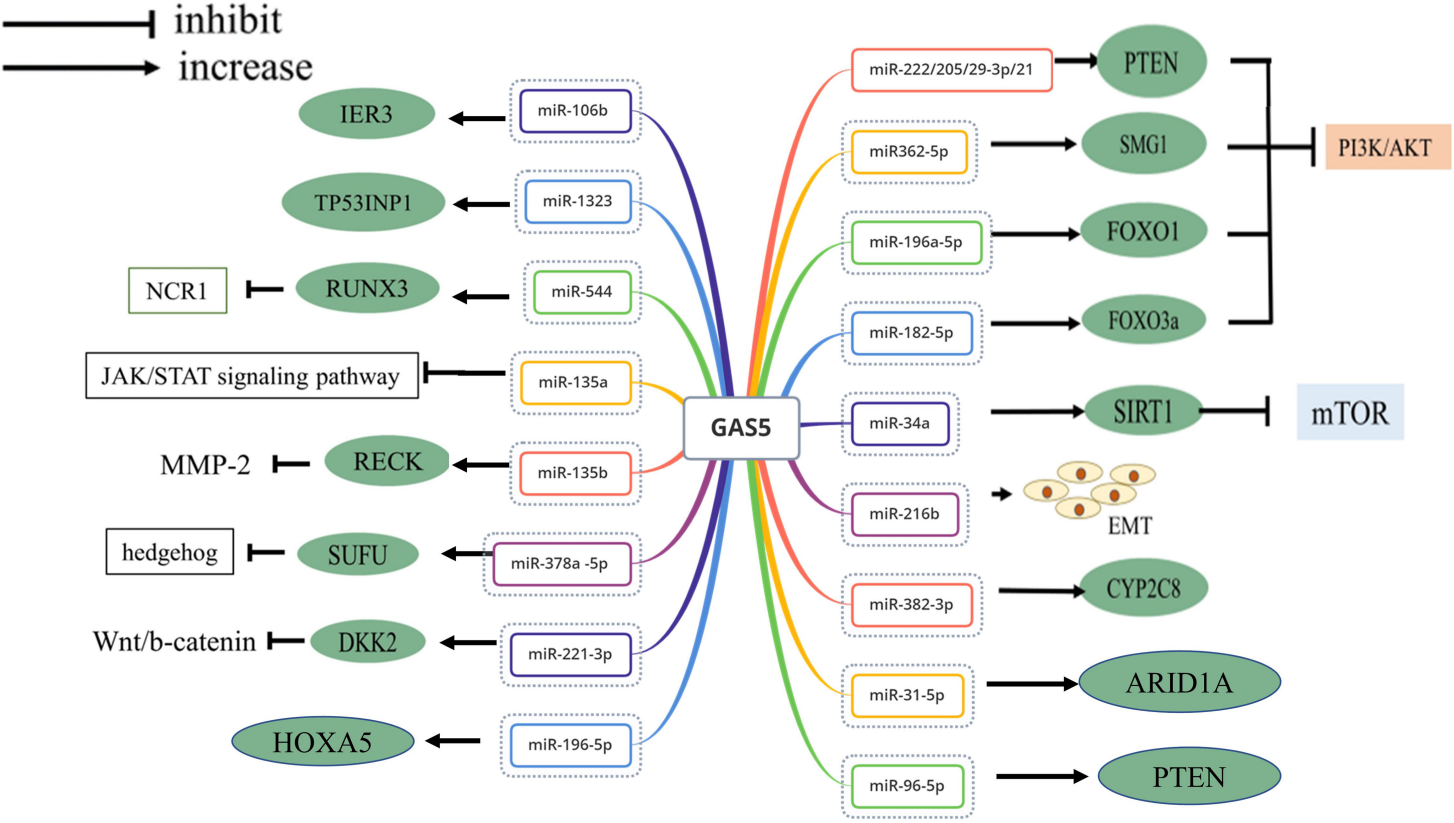
Network diagram of GAS5 adsorption of mi-RNA.

**Figure 3:**
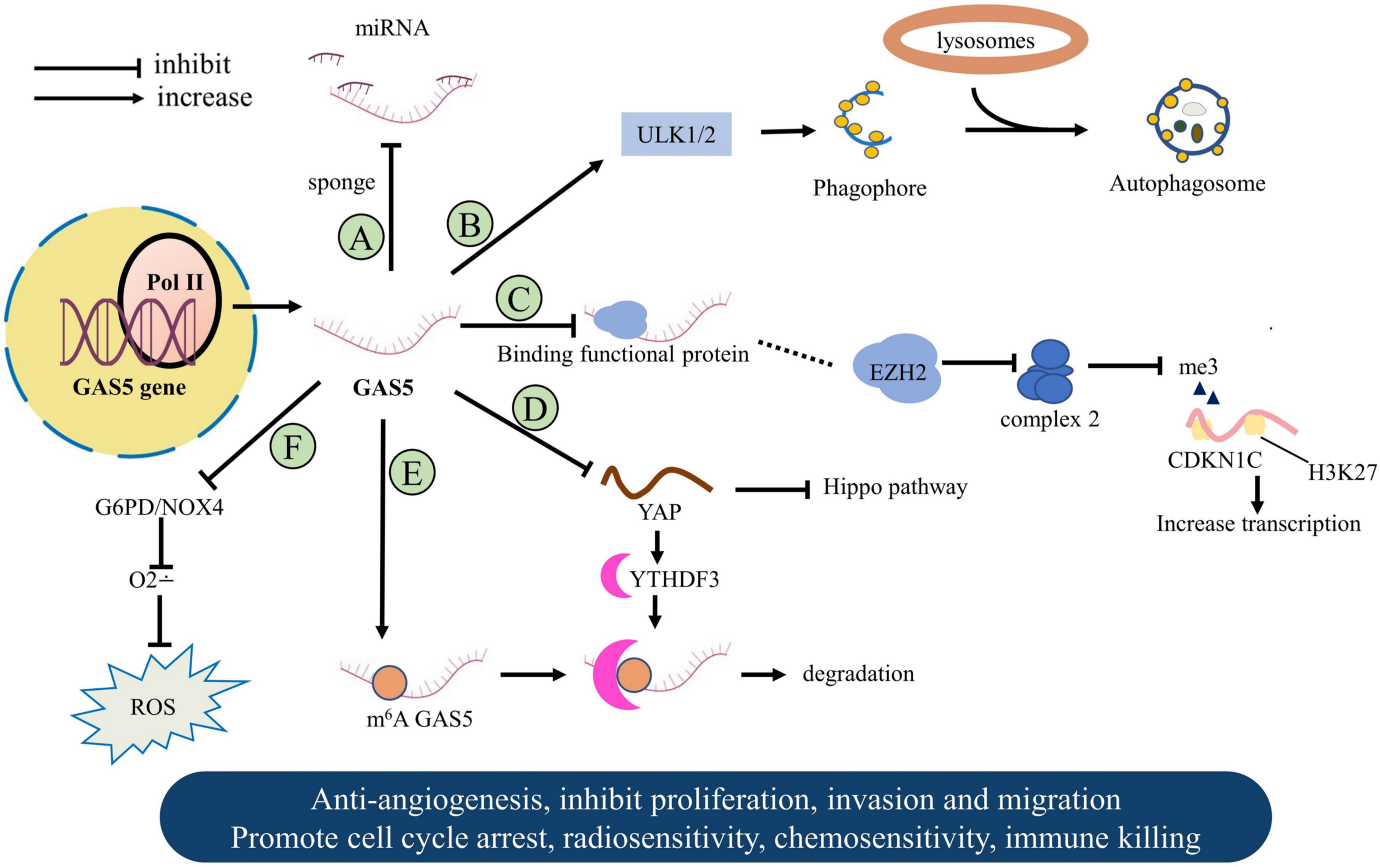
The molecular mechanism of GAS5 in a variety of human tumors. **(A)** GAS5 acts as a sponge to bond miRNA to suppress cancer. **(B)** GAS5 promotes the formation of autophagy in breast cancer. **(C)** GAS5 combines functional proteins to promote tumor suppressor gene expression. **(D)** GAS5 bind directly to WW domain of YAP in Colorectal cancer. **(E)** Methylated GAS5 is regulated downstream to form a negative feedback loop. **(F)** GAS5 changes the level of intracellular oxidative stress to maintain cell growth arrest.

The lncRNA GAS5 evolved various strategies to regulate gene expression as vital tumor proliferation suppressor. GAS5 acts as a “sponge-like” to interact with miRNA molecules (**Figure 2**), binds to one or more functional proteins, and limits tumor angiogenesis. Other related mechanisms are the tumor microenvironment affected by intracellular oxidative stress and immune escape (**Figure 3**). In this part, we will focus on the latest discoveries and various features of GAS5 that regulate malignant tumors.

### Breast Cancer

Asghar et al. (17) found that the expression of GAS5 in breast cancer (BC) tissues was less compared to the control group, and the expression of the younger group (< 45 years) was less than that found in the older group (> 45 years). The action mechanism indicated that GAS5 combined with miR-216b regulates the epithelial-mesenchymal transition of breast cancer tissues, promotes apoptosis, and weakens the invasion ability (18).

Autophagy refers to the process in which double-membrane vesicles wrap intracellular proteins or damaged organelles and integrate into lysosomes to decompose (19). The autophagic progress may play a dual role in the complex tumor microenvironment (20). Unc-51-like kinase 1 (ULK1) plays an active role in the initiation of autophagy. ULK1 and its homolog ULK2 both have been reported to induce autophagy and inhibit tumor progression. GAS5 is positively related to ULK1/2, and autophagy induction promotes autophagosome formation in BC. This can limit malignant behaviors such as proliferation, invasion, and formation of tumors (21).

Increasing evidence has shown that GAS5 is also involved in regulating thesensitivity of BC to chemotherapy drugs. As a member of the dickkopf family, dickkopf 2 (DKK2) regulates the Wnt/β-catenin signaling pathway by binding to the lipoprotein receptor related protein 5/6, a component of the Wnt receptor complex. GAS5 binds miR-221-3p to reduce the latter’s expression and increases DKK2 expression, further repressing the Wnt/β-catenin signaling pathway, thus reversing resistance to ADR (22). Similar studies by Gu et al. (23) also showed that GAS5 overexpression can act as a molecular sponge binding with miR-222, upregulating its endogenous target phosphatase and tensin homologs (PTEN), silencing the AKT/mTOR signaling pathway, and increasing the sensitivity of BC to tamoxifen.

Triple-negative BC (TNBC) is a unique subtype of breast cancer identified after the lack expression of the estrogen receptor, progesterone receptor, and epidermal growth factor receptor 2. TNBC is highly invasive and resistant to chemotherapy, and therefore it is considered the most aggressive and fatal type of BC. There is a study shows that GAS5 inhibits miR-196a-5p expression and its downstream target FOXO1 expression increases, which in turn inhibits PI3K/AKT phosphorylation, thus limiting TNBC progression (24). Another study discovered that overexpressed GAS5 could increase the expression level of SUFU by sponging miR-378a -5p in TNBC cells, resulting in enhanced apoptosis of cells resistant to paclitaxel (PTX) and cisplatin (CIS) (25).

### Lung Cancer

Non-small cell lung cancer (NSCLC) is the most common type of lung cancer. The intrinsic level of GAS5 is downregulated in NSCLC. This downregulation is inversely related to poor clinicopathological factors. GAS5 significantly inhibits the expression level of miR-205 in NSCLC, and miR-205 has been shown to interact with PTEN (26). Furthermore, Xue et al. (27) revealed that GAS5 remarkably inhibited miR-135b expression, increasing the sensitivity of NSCLC to radiotherapy.

Due to their different contents, exosomes are currently extensive studied as emerging players in intercellular information transmission. Poulet et al. (28) found that the low expression of exosomal GAS5 in serum from NSCLC patients was positively associated with advanced tumor–node–metastasis (TNM) stages and larger tumor diameter than healthy controls. In human umbilical vein endothelial cells, GAS5 in exosomes has been shown to bind competitively with miR-29-3p, changing PTEN and blocking the PI3K/AKT pathway (HUVEC). As a result, exosomal GAS5 might affect the control of NSCLC angiogenesis (29).

### Prostate Cancer

Recently, programmed cell death 4 (PDCD4) affects the expression of various oncogenes and may be positively correlated with prostate cell differentiation. Single-nucleotide polymorphisms (SNPs) were shown to affect the expression level of the lncRNA in which it is located. The two SNPs (rs55829688 T > C and rs145204276 INS > DEL) in GAS5 have been shown to upregulate GAS5 expression by methylating its promoter, and then GAS5 binds miR-21 and miR‐2184 directly as a sponge, targeting PTEN, PDCD4, respectively, further inducing cell apoptosis in Prostate Cancer (PCa). The aforementioned findings mentioned above indicate that GAS5 may be a novel prognostic marker of PCa (30).

### Colorectal Cancer

GAS5 expression was associated with the clinicopathological characteristics of CRC, and patients with high GAS5 expression had a smaller tumor diameter and early TNM staging. GAS5 levels are significantly reduced compared to normal controls, causing the proliferation, migration, and invasion of cancer tissues (31). Cheng et al. (32) reported that FOXO3a inhibits the phosphorylation of the PI3K/AKT signaling pathway as a pro-apoptotic transcription factor, GAS5 directly adsorbs miR-182-5p, increases the expression of the downstream target FOXO3a, which causes the proliferation of CRC and promotes apoptosis.

Sirtuin 1 (SIRT1) is a type III nuclear deacetylase, which is upregulated in CRC and negatively regulates the expression level of mTOR, which is negatively correlated with the level of intracellular macroautophagy activity. Separate studies have demonstrated that GAS5 directly adsorbs miR-34a like a sponge, releasing its inhibition of SIRT1 expression, further inhibiting the level of macroautophagy activation and inducing CRC apoptosis. Furthermore, the 5’-TOP sequence located in the GAS5 exon controls its transcription. Overexpression of miR-34a forms a negative feedback regulation loop through the SIRT1/mTOR/GAS5 axis, which may explain why GAS5 - mediated autophagy in CRC is in a relatively balanced state. Thus, it plays an anti-apoptotic role in cancer progression and provides a new perspective on the molecular mechanism of CRC (33).

A central downstream sensor of the Hippo pathway is phosphorylated YAP, which can promote the progression of CRC. GAS5 binds the 171-263 amino acid region (WW domain) of YAP directly to promote ubiquitination and degradation of the latter, thus inhibiting YAP signaling. Further research indicated that YTH-domain family member 3 (YTHDF3) acts as an N^6^-methyladenosine (m^6^A) reader, considered as a new YAP target. YTHDF3 selectively binds to this structure and promotes the degradation of GAS5 through methylation dependence, forming a negative feedback loop. Therefore, YAP activates ectopically and promotes the malignant behavior of CRC in the absence of GAS5. The stable expression level of GAS5 is of great significance in limiting the progression of CRC (34).

### Malignant Melanoma

Previous reports have indicated that malignant melanoma (MM) is characterized by aggressiveness and early metastasis, and its pathogenesis and development are related to intracellular oxidative stress (35). Reactive oxygen species are oxygen-containing derivatives composed of highly unstable oxygen free radicals and are elevated in tumor cells. ROS has been indicated act as a cancer promoters due to the abnormal process of proliferation, apoptosis, and angiogenesis effect (36). Chen et al. reported that GAS5 diminished remarkably in advanced melanoma. GAS5 overexpression induces cancer cells to stay in the G0/G1 phase and inhibits the expression of G6PD and NOX4, further leading to a decrease in O2∸. The above changes reduce the level of reactive oxygen species and destabilize the redox balance in MM cells (37). The work of Xu et al. (35) showed that GAS5 participates in facilitating tumor repression by directly binding EZH2, a member of the Polycomb inhibitory complex 2, further reducing lysine 27 trimethylation (H3K27me3) of histone H3, enhancing the expression of the target gene CDKN1C. Taken together, these processes mentioned above accelerate intracellular oxidative stress and inhibit the vitality of MM.

### Bladder Cancer

Studies by Avgeris et al. (38) encouraged the notion that bladder cancer (BlCa) expressing lower levels of GAS5 than that in adjacent normal tissues, and GAS5 expression in invasive (T1-T4) tumors was significantly attenuated than in superficial Ta tumors. One mechanism shows that overexpressed GAS5 acts as a sponge *via* binding with and downregulation of miR-21, amplify-function mutations in PTEN, and promote tumor apoptosis; knocking out GAS5, in contrast, increases the proportion of S and G2 cancer cells, thereby promoting proliferation (39). In bladder transitional cell carcinomas (BTCC), GAS5 expression is reduced and the patient’s prognosis worsens. Whereas overexpression of GAS5 inhibits the anti-apoptotic gene Bcl-2, upregulation of Bcl-2 reversed the inhibitory effect of GAS5 on doxorubicin-resistant BTCC. Accordingly, GAS5 contributes to the development of drug resistance (40).

### Hepatic Carcinoma

The low expression of GAS5 may reflect the poor survival rate of patients with hepatocellular carcinoma (HCC). The cytochrome P450 family 2 subfamily C member 8 (CYP2C8) is well recognized for inhibiting cell proliferation growth in HCC. GAS5 induces apoptosis by sponging miR-382-3p and upregulating the expression of CYP2C8 proteins (41). Reversion-inducing cysteine-rich protein with Kazal motifs (RECK) is an anti-metastatic gene cloned from NIH3T3 cells, which encodes extracellular proteins and restricts the metastasis of HCC by blunting the effects of matrix metalloproteinases (MMPs). Notably, the invasive ability of the tumor may be eliminated through a combination of upregulated GAS5 and miR-135b, exhibiting increased RECK, and the expression and activity of MMP-2 are insufficient (42). The tumor protein p53-inducible nuclear protein 1 (TP53INP1) can induce apoptosis as a stress-induced gene by regulating the p53 and p73 pathways and is attenuated by miR-1323. The apoptotic machinery is composed of GAS5 that binds to miR-1323, directly stimulating the function of TP53INP1 as a barrier to the pathogenesis of HCC (43).

Evidence began to accumulate in GAS5-mediated regulation of the immune system. For example, the activating receptor NKp46 encoded by the natural cytotoxicity receptor 1 (NCR1) can be expressed in NK cells; this work limits tumor growth and metastasis by improving IFN-c release, while Runt-related transcription factor 3 (RUNX3) acts on the NCR1 promoter in NK cells and has demonstrable increases in function. Studies on controlling mechanisms suggest that GAS5 negatively regulates miR-544, activating RUNX3 expression, and increasing the immune killing of HCC through the RUNX3/NCR1/NKp46 axis. As such, these may represent a potential hallmark for the treatment of liver cancer (44).

### Gastric Cancer

Li et al. (45) found that GAS5 binds miR-222 through an endogenous sponge mechanism that acts on PTEN, which modulates the classical signaling pathway PI3K/Akt/mTOR and subsequently inhibits GC proliferation. In addition, Liu et al. (46) reported that tumor size, lymph node metastasis, and TNM staging in patients were negatively associated with GAS5 gene amplification. Higher GAS5 expression increases protein expression of p53 and its target gene KAI1/CD82, and helps extend the half-life of p53, which correlates with poor metastasis capacity with GC.

Moreover, a study demonstrated that the progression of GC is driven by rs145204276 of GAS5, patients with allele del of rs145204276 located in the promoter region of GAS5, would result in a smaller tumor size and a lower risk of GC progression (47).

### Cervical Cancer

Cancer cells with low express-mutated GAS5 in cervical tissue represent a survival advantage; on the contrary, its overexpression weakens the malignant biological behavior of cervical cancer (CC). The underlying mechanism suggests that GAS5 has shown a downside function in regulating miR-135a, silencing the JAK/STAT signaling pathway (48). Besides, Yang et al. uncovered that elevated GAS5 expression limits the growth and metastasis of CC by interacting with the epithelial-mesenchymal transition (49). In drug resistance research, GAS5 overexpression was remarkably associated with inhibition of miR-21 expression and significantly increased the expression level of PCDC4 and TIMP3, which activate sensitivity to cisplatin in CC cells (50). Similarly, upregulation of IER3 shortens the cycle of keratinocytes and enhances the sensitivity of CC cells to radiation. Mechanically, GAS5 can decrease miR-106b expression and raise the expression level of IER3 (51).

### Thyroid Cancer

In thyroid cancer, GAS5 expression is decreased in poor phenotypes, suggesting that patients are at increased risk of recurrence (52). A recent study suggests that the suppressor of morphogenesis in genitalia 1 (SMG1) inactivates the AKT/mTOR signaling pathway in various cancers. Remarkably, GAS5 overexpression competitively binds miR362-5p and therefore supports an active effect of SMG1, facilitating apoptosis of 131I-resistant TC *via* the dephosphorylated Akt/mTOR signal. This mechanism almost indicates that GAS5 is involved in the regulation of radiotherapy sensitivity with TC cells (53).

### Ovarian Carcinoma

Both GAS5 and ARID1A (AT-rich interactive domain 1A) are underexpressed in ovarian clear cell carcinoma (OCCC). Aberrant loss of ARID1A expression in most patients with OCCC, resulting in loss of the expression of BRG-associated factor 250a (BAF250a) protein, is associated with a poor prognosis in OCCC. Further research indicated that GAS5 could binds with miR-31-5p and significantly upregulate ARID1A to suppress the viability and invasion of OCCC cells (54). Recently, GAS5 overexpression has been shown to inhibit the proliferation and migration ability of ovarian carcinoma (OC). Mechanically, GAS5 binds miR-96-5p as a ceRNA. Further research confirmed that miR-96-5p specifically decreased the expression of PTEN (55). Similarly, Zhao et al. demonstrated that GAS5 could act as a key target of miR-196-5p to downregulating the expression of HOXA5. Abnormal deletion of HOXA5 is implicated in proliferation and apoptosis in OC. Thus, the GAS5/miR-196a-5p/HOXA5 signaling pathway may be a novel lncRNA-based strategy to improve the prognosis of OC (56).

### Mesothelioma

Malignant pleural mesothelioma (MPM) is a rare tumor caused primarily by exposure to asbestos. A previous study indicated that the expression level of GAS5 is lower in MPM compared to normal mesothelial cells. Interestingly, decreased GAS5 increases the expression of genes that respond to glucocorticoid receptor in MPM. Studies have shown that glucocorticoids play a vital role in the progression of MPM, GAS5 may inhibit the transcription of glucocorticoid-responsive genes through its structural glucocorticoid receptor response element (57).

## Conclusions and Future Perspectives

We have sought to find an overall understanding of the mechanism in the GAS5-mediated cancer process (**Table 1**), which can offer a perspective on revealing the complex biology of cancer. As illustrated in this review, the lncRNA GAS5 has been dysregulated in several types of cancer and is reported to significantly decrease in most types of cancers. These works are closely paving the way for a GAS5-related clinical phenotype and a poor prognosis in cancer patients. The molecular mechanism clarified that GAS5 interacted with several miRNAs to deliver suppressive information, resulting in uncontrollable interactions of many signaling molecules and pathways, including the SUFU signaling, Wnt/β-catenin signaling, and PI3K/AKT signaling pathways. Other GAS5 mechanistic models have identified that cancer cells may further deteriorate due to the dysregulation of autophagy, oxidative stress, and immunity. Furthermore, GAS5 can also regulate sensitivity to chemoradiation, demonstrating that GAS5 has potential clinical application value for tumor therapy.

**Table 1:** LncRNA GAS5 in human cancers.

Cancer types	Regulatory molecules	Signal	Functional role	Years	References
Breast cancer	GAS5/ miR-216b/ EMT		Proliferation, apoptosis, migration, invasion	2020	(18)
	GAS5/ULK1、 ULK2/autophagy		Proliferation, invasion	2020	(21)
	GAS5/miR-221-3p/DKK2	Wnt/β-catenin	Proliferation, chemoresistance	2020	(22)
	GAS5/miR-222/PTEN	PI3K/AKT	Proliferation chemoresistance	2018	(23)
	GAS5/miR-196a-5p/FOXO1	PI3K/AKT	Proliferation, migration, invasion, apoptosis	2018	(24)
	GAS5/ miR-378a-5p/SUFU	Hedgehog	Proliferation, chemoresistance	2020	(25)
Lung cancer	GAS5/miR-205/PTEN/PI3K/AKT/hTERT	PI3K/AKT	apoptosis , cell cycle	2016	(26)
	GAS5/miR-135b		Proliferation, radiosensitivity	2017	(27)
	GAS5/miR-29-3p/PTEN/	PI3K/AKT	Proliferation, angiogenesis	2019	(28)
Prostate cancer	GAS5/miR-21/PDCD4/PTEN	PI3K/AKT	Proliferation, migration, invasion, apoptosis	2019	
Colorectal cancer	GAS5/miR-182-5p/FOXO3a	PI3K/AKT	Proliferation, apoptosis	2018	(31)
	GAS5/miR-34a/SIRT1/mTOR/ autophagy		Proliferation, apoptosis	2021	(32)
	GAS5/YAP/YTHDF3	Hippo	Proliferation, migration, invasion	2019	(33)
Malignant melanoma	GAS5/EZH2/H3K27me3/ CDKN1C/ROS		Proliferation, apoptosis	2020	(34)
	GAS5/ G6PD、NOX4/ROS		Proliferation, cell cycle, apoptosis	2019	(36)
Bladder cancer	GAS5/ miR-21/ PTEN		Proliferation, apoptosis	2020	(38)
	GAS5/ Bcl-2		Proliferation, chemoresistance	2017	(39)
Hepatic carcinoma	GAS5/ miR-382-3p/ CYP2C8		Proliferation, apoptosis	2020	(40)
	GAS5/ miR135b/ RECK/ MMP-2		Proliferation, migration, invasion,	2019	
	GAS5/ miR-1323/ TP53INP1		Proliferation, migration, invasion, apoptosis	2019	(41)
	GAS5/miR-544/RUNX3/ NCR1/NKp46		Proliferation, immune killing	2019	(42)
Gastric Cancer	GAS5/miR-222/PTEN/PI3K/ Akt/mTOR	AKT/mTOR	Proliferation	2017	(43)
	GAS5/p53/KAI1/CD82		Proliferation, migration, invasion,	2020	(44)
	rs145204276/GAS5		Proliferation, susceptibility	2018	(45)
Cervical cancer	GAS5/ miR-135a	JAK/STAT	Proliferation, migration, invasion,	2020	(46)
	GAS5/ STAT3/ miR-21/ PCDC4、TIMP3	STAT3	Proliferation, chemotherapy	2019	(48)
	GAS5/miR-106b/IER		Proliferation, radiosensitivity	2019	(49)
Thyroid cancer	GAS5/miR362-5p/SMG1 Akt/mTOR	AKT/mTOR	Proliferation, chemotherapy	2020	(51)
Ovarian carcinoma	GAS5/miR-31-5p/ARID1A/ BAF250a		Proliferation, invasion	2021	(52)
	GAS5/miR-96-5p/PTEN	AKT/mTOR	Proliferation, migration	2021	(53)
	GAS5/ miR-196-5p / HOXA5		Proliferation, apoptosis	2018	(54)
Mesothelioma	GAS5/glucocorticoid responsive genes/leucine-zipper and serum/glucocorticoid-regulated kinase-1	Hedgehog/PI3K/mTOR	Growth arrest	2014	(55)

Many studies highlight that GAS5 acts as a sponge to regulate various miRNAs. However, the specific mechanisms are rarely discussed. Previous research predicted that GAS5 has a miR-1323 binding site and demonstrated it by luciferase reporter assays (43),which indicated that GAS5 base pair has a particular complementary sequence with miRNA. Additionally, we predict that RNA-induced silencing complex (RISC) may play an important role in binding GAS5 and miRNA, a primary miRNA transcript (pri-miRNA) that undergoes cleavage, transport, and cleavage into mature miRNAs, which are finally incorporated into the RISC. Zhang et al. detected that over expression of GAS5 induces the reduction of mature miR-21. However, pri-miR-21 and pre-miR-21 are not affected by GAS5. Therefore, GAS5 may regulate miRNAs in the RISC complex *via* a post-transcriptional pathway. Furthermore, they detected that both GAS5 and miR-21 are enriched in AGO2 - an essential part of RISC (58). The combination of GAS5 and miR-21 in RISC may suggest why GAS5 easily binds miRNA, but the related mechanism needs further research to verify.

In the present review, there is an essential general trend to explore the interaction between GAS5 and intracellular oncogenes. Such observations suggest that GAS5 was portrayed as a tumor suppressor in the cancer cell cycle. It is also observed that some activators partly regulate the level of GAS5, and this may provide a foundational theoretical basis for explaining the occurrence and development of malignant tumors. However, the interaction between GAS5 and the molecules presents diversity and complexity in the tumor microenvironment, and a complete understanding of the mechanism network is still far beyond our reach. Therefore, further searching for highly sensitive and specific biomolecular markers that interact with GAS5 exists is of great clinical research value.

## Author Contributions

GL and TW contributed to conceptualization and full-text writing. XG, ZH and WN contributed to critical reading of the manuscript. All authors read and approved the final manuscript.

## Conflict of Interest

The authors declare that the research was conducted in the absence of any commercial or financial relationships that could be construed as a potential conflict of interest.

## Publisher’s Note

All claims expressed in this article are solely those of the authors and do not necessarily represent those of their affiliated organizations, or those of the publisher, the editors and the reviewers. Any product that may be evaluated in this article, or claim that may be made by its manufacturer, is not guaranteed or endorsed by the publisher.
